# Does choice change preferences? An incentivized test of the mere choice effect

**DOI:** 10.1007/s10683-021-09728-5

**Published:** 2021-08-15

**Authors:** Carlos Alós-Ferrer, Georg D. Granic

**Affiliations:** 1 Zurich Center for Neuroeconomics (ZNE), Department of Economics, University of Zurich, Zurich, Switzerland; 2 Department of Applied Economics, Erasmus University Rotterdam, Rotterdam, The Netherlands; 3 Department of Marketing, University of Antwerp, Antwerp, Belgium

**Keywords:** Mere choice effect, Preference change, Stability of preferences, C91, D01, D91

## Abstract

Widespread evidence from psychology and neuroscience documents that previous choices unconditionally increase the later desirability of chosen objects, even if those choices were uninformative. This is problematic for economists who use choice data to estimate latent preferences, demand functions, and social welfare. The evidence on this *mere choice effect*, however, exhibits serious shortcomings which prevent evaluating its possible relevance for economics. In this paper, we present a novel, parsimonious experimental design to test for the economic validity of the mere choice effect addressing these shortcomings. Our design uses well-defined, monetary lotteries, all decisions are incentivized, and we effectively randomize participants’ initial choices without relying on deception. Results from a large, pre-registered online experiment find no support for the mere choice effect. Our results challenge conventional wisdom outside economics. The mere choice effect does not seem to be a concern for economics, at least in the domain of decision making under risk.

**Supplementary Information:**

The online version contains supplementary material available at 10.1007/s10683-021-09728-5.

## Introduction

The ability to recover preferences from choice data, and subsequently predict choices from preferences, is fundamental for economic analysis. The revealed preference approach (Samuelson, 1938, 1948; Houthakker, 1950; Arrow, 1959; Richter, 1966) essentially views preferences as nothing more than organizing schemes reflecting both observed and predicted choices. More recent accounts have shown that the ‘spirit’ of the revealed preference approach can be conserved under more general conditions. For example, the literature on stochastic choice and random utility models explicitly incorporates variability in choice—a key observation in real-world choice data (Tversky, 1969; Hey and Orme, 1994; Agranov and Ortoleva, 2017)—by adding random components to true, underlying preferences (McFadden, 1974, 2001). Provided that stable mechanisms govern choice variability, true preferences can still be recovered (e.g., Apesteguía and Ballester, 2018; Lu and Saito, 2020; Frick et al., 2019; Alós-Ferrer et al., 2021). In practice, choice data is universally used to estimate latent and derived concepts ranging from utility functions and risk attitudes to demand functions and social welfare (e.g., Harsanyi, 1955; Koopmans, 1960; Afriat, 1967; Varian, 1982; Andreoni and Miller, 2002; Cox et al., 2008; Deb et al., 2014, among many others). The use of choice data, however, entails an implicit but rarely-discussed assumption: stability. Predicting future choices from preferences which themselves are estimated from past choices is only warranted as long as economic agents display well-defined and stable choice patterns (or, at least, stable mechanisms governing choice variability) in the relevant time frame.

Worryingly, the assumption of stable choice patterns (deterministic or stochastic) is at odds with fundamental theories in psychology, which postulate that choices can create and alter preferences (Festinger, 1957; Bem, 1967a, b; Slovic, 1995; Simon et al., 2004; Ariely and Norton, 2008). That is, the mere act of choice, even when no new information is revealed by or after the choice, can lead to fundamental changes in preferences, so that we do not only “choose what we like,” but mechanically also “like what we choose.” Empirical support for such feedback loops between choices and preferences appears to be widespread (Egan et al., 2010; Sharot et al., 2010; Nakamura and Kawabata, 2013; Johansson et al., 2014). These alleged preference changes occur within the time span of a few minutes and in the absence of any new, choice-relevant information. They are therefore fundamentally problematic for economics. If such effects extend to economic choices, every choice-based preference elicitation procedure bears the potential to interfere with the very concept it ought to measure. Observed economic choices may then permanently lag behind current preferences, and standard economic applications estimating utilities, demand, and social welfare may be systematically biased.

In light of its potential consequences, it is of paramount importance to investigate the economic validity and significance of this *mere choice effect*. Evidence from psychology is insufficient to settle the question, due to difficulties with the experimental paradigms applied in that literature (see next section), the hypothetical nature of choices in such studies, and the non-economic nature of the alternatives they study. This paper undertakes the endeavor of establishing the validity of the mere choice effect (preference change due purely to the act of choice) *relying on incentivized choices*. We develop a parsimonious experimental design that allows researchers to isolate the effect of mere, uninformative choices on future choices in an economically-relevant domain (binary monetary gambles or lotteries). In essence, our experimental design first presents participants with two choice options (lotteries), but, crucially, the experimenter randomly determines whether a certain choice option is transparently inferior or superior (through stochastic dominance). As choices are incentivized, it is in the best interest of participants to choose the objectively superior option and hence follow the pre-determined, randomized choice patterns. Further choices in the experiment then test for preference change in favor or against the previous options. In this way, the design effectively randomizes uninformative (mere) choices. We hereby solve typical issues encountered in the existing literature: unreliable preference measures, hypothetical bias, and deception (we will elaborate on these issues in the next section).

This paper reports the results of a large-scale, preregistered online experiment (the paper was evaluated at the journal previous to data collection) relying on the basic design described above. The mere choice effect was assessed by measuring whether merely-chosen options were *subsequently* chosen more often than merely-rejected ones. The results hence allow us to establish whether or not the mere choice effect is relevant for economics and whether or not it is warranted to maintain a unidirectional link between choices and preferences in the domain we study. Hereby, we contribute to a stream of literature that discusses the possibility of past experiences shaping future preferential choices. For example, the literature on preference discovery postulates that decision makers do not know their true tastes until they (incompletely) discover them through consumption experience (Plott, 1996; Braga and Starmer, 2005; Delaney et al., 2020). Naturally, past choices are a vital input source for the discovery process. Frick et al. (2019), on a related note, discuss in their concluding remarks that their dynamic random utility framework could accommodate endogenously evolving preferences (as a function of the agent’s past consumption level). This could take the form of habit formation or simply reflect the fact that past consumption provides payoff-relevant information. In contrast, in this paper we aimed to establish whether past choices affect future choices even when there is nothing to be learned from them. A possible mechanism would be that decision makers, to some extent, are used (or hard-wired) to learn from past choices and they mistake uninformative choices for informative ones. This could result in “decision inertia” as studied by Alós-Ferrer et al. (2016b) even in cases where objectively superior options are available (see also Jung et al., 2019). For example, Cerigioni (2017) studies how past exposure to a choice option creates inertia or stickiness towards the exposed option. This stickiness, known as the mere exposure effect (Zajonc, 1968,, 2001), may explain behavioral regularities like the status-quo bias, and could at least partially drive the mere choice effect (we will discuss this latter possibility below).

We committed to our conditional conclusions prior to data collection. In case supporting evidence for the mere choice effect would have been found, the intention of our work was to contribute to the development of better and more predictively-accurate preference elicitation methods. For example, if preference change followed regular patterns, standard elicitation procedures could be corrected by taking into account quantitative predictions about the expected magnitude of preference change. However, our experiment found no supporting evidence for the mere choice effect. Merely-chosen and merely-rejected lotteries were subsequently chosen with almost identical frequencies, which in turn were almost identical in magnitude to a baseline measurement. Since our study had sufficient power, the conclusion is that the effects reported in psychology are likely to be too small for economic choices to merit sparking a major reevaluation of economic methods.

The remainder of the paper is structured as follows. Sect. 2 reviews the existing literature on choice-induced preference change and briefly discusses the main theory underlying the effect. Sect. 3 presents our experimental design, including the derivation of our main hypothesis and the power analysis. Sect. 4 presents the statistical analyses (as planned before data collection and actually carried out) and discusses the interpretation of the results and Sect. 5 concludes. Additional results and supplementary experimental materials (experimental instructions and screenshots) are presented in the online appendix.

## Literature review: choice-induced preference change

Psychological theories that explain how choices can create preferences often draw an analogy between how we make inferences about others’ preferences and how we make inferences about our own preferences (Bem, 1967a, b; Ariely and Norton, 2008). As we cannot fathom what others feel and think, we infer their preferences and beliefs by what we can observe: their behavior. If we observe a stranger on the street giving money to a homeless person, we infer that the stranger is altruistic. Analogously, if our own preferences are vague, imprecisely formulated, or incomplete, we cannot fathom what we ourselves feel and think. Thus, we infer our own preferences from what we can observe: our own past behavior. Imagine a consumer standing in front of a drug-store shelf filled with many shampoo brands. One particular brand catches her eye. She is not quite certain of whether she likes the brand or not, but remembers buying it in the past. She deduces that there must have been a good reason for that decision. Being a rational consumer, the shampoo must have fulfilled her needs. She infers that she likes the shampoo and buys it again. This line of reasoning can lead us astray because memory often inaccurately captures hedonic experiences. For example, it is well understood that unrelated situational factors can impact behavior and that we are not always aware of their influence (Slovic, 1995; Ariely et al., 2003; Ariely and Norton, 2008; see, however, Fudenberg et al., 2012 and Maniadis et al., 2014). Maybe the consumer correctly remembers buying the shampoo, but forgets having been in a rush that day, or that the shampoo was part of a promotional deal. In that case, her self-inference process was based on an inaccurate recollection of a past event. This is the logic behind the mere choice phenomenon, with the only caveat that, in psychology, processes of preference change are assumed to happen subconsciously. Uninformative (mere) choices can serve as input factors for the self-inference process, which itself may then lead to wrongly imputed preferences.^1^

Most of the relevant evidence on preference change in psychology has been collected using the following three-stage setup. In stage 1, participants rate or rank certain objects, like artistic paintings, on their desirability. In stage 2, they are asked to make a choice between two previously-rated objects. Participants are led to believe that they have made a free choice, but, in reality, researchers use some form of deceptive technique to manipulate choice and randomly determine what was chosen and rejected, e.g. alleged subliminal choice (Sharot et al., 2010). In the third and final stage, objects are rated or ranked again. Preference change is measured by comparing how much chosen objects have increased in self-reported desirability relative to rejected objects. The typical finding is that chosen objects are reevaluated upwards and non-chosen ones are reevaluated downwards, even if choices were randomly assigned. If preferences are stable, one should have observed no changes in desirability.

In spite of an apparently-overwhelming body of evidence, economists should be skeptical about the relevance of the mere choice phenomenon as currently established. First, the extant literature typically studies the effect of past choice on future desirability measures, e.g., liking ratings or rankings (Sharot et al., 2010; Nakamura and Kawabata, 2013). In economics, the most relevant data source is actual choices, and preferences are just binary relations organizing those choices, which decision makers might or might not have conscious access to. Whether (typically unincentivized) desirability measures proxy choice data sufficiently well is not self-evident (Cason and Plott, 2014). Hence, it is important to establish the mere choice effect on actual, subsequent choices and not only self-reported desirability scales. Second, the available experimental evidence exclusively investigates preferences in hypothetical choice scenarios over ill-defined options, which do not reference all preference-relevant option dimensions (Egan et al., 2010). Examples include hypothetical holiday destinations described by their destination names only, or the attractiveness of human faces (Sharot et al., 2010; Johansson et al., 2014). In such cases, behavior might be extremely noisy and easily swayed by irrelevant factors (Murphy et al., 2005; Fudenberg et al., 2012). The hypothetical bias identified in related domains casts doubts on whether the behavior observed in such paradigms is informative enough to study preference change (Hertwig and Ortmann, 2001; Murphy et al., 2005; Harrison and Rutström, 2008). Third, the existing literature has adopted research designs that deceive participants to achieve experimental control. For example, experimenters give wrong feedback about past choice using card tricks (swapping choices) or present cover stories about subliminal decision making and have a computer prompt a random choice (Sharot et al., 2010; Nakamura and Kawabata, 2013; Johansson et al., 2014). Deception is obviously inappropriate in experimental economics and, through lab reputation, would render any incentivized design ineffective. In summary, it remains unresolved whether actual choices, in contrast to perceived and make-believe choices, lead to preference change.

It needs to be pointed out that a large part of the literature on choice-induced preference change in psychology has studied a related but different question, namely whether and how choices involving some sort of tradeoff change preferences (Brehm, 1956; Harmon-Jones and Mills, 1999; Shultz et al., 1999; Jarcho et al., 2011; Alós-Ferrer et al., 2012; Izuma and Murayama, 2013). The dominant theory behind such effects is cognitive dissonance (Festinger, 1957; Akerlof and Dickens, 1982). In a nutshell, the underlying hypothesis is that a choice involving tradeoffs creates dissonance (psychological discomfort). Dissonance arises, because the chosen option has some negative characteristics and the rejected option has some positive ones (i.e., tradeoffs). The decision makers unconsciously reduce this dissonance by adjusting their preferences. Hereby, they reevaluate the chosen options up and rejected ones down. However, it has been recently shown that the experimental paradigm which has guided the development of this literature for over 50 years is regrettably flawed. It contains a statistical bias that can result in apparent preference change even if participants have stable preferences (Chen and Risen, 2010; Izuma and Murayama, 2013; Alós-Ferrer and Shi, 2015). Although some improved designs have been proposed (e.g., Alós-Ferrer et al., 2012), how the effect of tradeoff choices in economically-relevant domains could be studied remains an unresolved issue at the time of writing. Although beyond the scope of the current paper, it would of course also be valuable for economics to understand if and when tradeoff choices change preferences. This work, however, concentrates on the mere choice effect, which more clearly isolates the possible effects of the act of choice on preferences.

Finally, it should be pointed out that the mere choice effect may be related to a general tendency in decision makers to develop a ‘preference’ for alternatives merely because they have been exposed to them. This tendency, know as the mere exposure effect, is robust and has been replicated across many domains (Zajonc, 1968; Bornstein, 1989; Monahan et al., 2000; Zajonc, 2001). It may be the case that exposure is asymmetric, stronger for chosen options than for non-chosen ones (see, e.g., Alós-Ferrer et al., 2016b; Cerigioni, 2017). In this view, preference changes are then not caused by the act of choosing, but by exposure alone. Returning to the example at the beginning of this section, imagine that the consumer remembers her reasoning process, but forgets what choice it led her to. Asymmetric mere exposure would nevertheless predict an increase in ‘preference’ for the chosen shampoo, at least against options which were not visible or available at the time. We will provide a critical discussion of how the mere exposure effect might affect our experiment and the results we find in Sect. 4.

## Experimental design and procedures

### Design and main hypothesis

We developed a novel experimental design that bypasses all of the critiques and difficulties mentioned above. First, we study the impact of past choices on subsequent ones, and hence our dependent variable are choices, the most relevant preference measure in economics. Second, we do so using lotteries. Lotteries have well-defined, objective, and economically-relevant characteristics (probabilities and monetary outcomes). This allows us to induce monetary incentives, which eliminates any potential hypothetical bias. Finally, we achieve control over initial choices without using any form of deception. To this end, we exploit the well-defined structure of lotteries. In our design, initial choices are made between a fixed target lottery, *a*, and a new lottery, *c*, which is constructed on the spot. We randomly determine at the participant-level whether the constructed lottery *c* is transparently inferior or superior monetary-wise to the target lottery *a*. Assuming only that participants prefer more money over less money, they should follow the randomly pre-determined choice patterns. If *c* is inferior, participants should choose the target lottery *a*. If *c* is superior, participants should reject *a*. We call these predicted choices *mere choices*, as they do not reveal any new information about the underlying preferences over lotteries. After mere choices, we subsequently elicit choices between the target lottery *a* and a fixed, not-previously-encountered third lottery *b*. Call this choice the *preference choice* (*a*, *b*). Crucially, preference choices involve tradeoffs and *a* is neither superior nor inferior to *b* in a dominance sense. The mere choice effect can now be measured precisely. If mere choices change the desirability of lottery *a*, we can expect lotteries *a* that were merely-chosen to be more attractive than comparable lotteries *a* that were merely-rejected. This in turn should impact the choice frequencies in preference-choices (*a*, *b*). Merely-chosen lotteries *a* should be chosen more often than merely-rejected lotteries *a* in preference-choices (*a*, *b*). We can formulate our main research hypothesis as follows:

**H1**: *Frequency*(*a* chosen over *b* $\mid$ *a* is merely-chosen) >

*Frequency*(*a* chosen over *b* $\mid$ *a* is merely-rejected)

### Procedures

**Table 1:** Sample demographics. *N* and $N\%$ represent absolute and relative frequencies, respectively. Percentages in $N\%$ columns were calculated excluding “Prefer not to disclose” (PNTD) answers. Percentages in UK% columns represent the most recent UK adult population figures taken from the UK Office for National Statistics and the OECD

	*N*	$N\%$	UK%
*Household income*			
Less than 19,000	199	25	17
19,000 to 31,999	197	25	28
32,000 to 47,999	157	20	24
48,000 to 63,999	125	16	14
64,000 or more	105	14	17
Prefer not to disclose (PNTD)	57	–	–
Total	840	100.0	100.0
*Highest education level*			
No academic or professional qualifications	7	1	7.9
Level-1	35	4	9.9
Level-2	68	8	15.7
Trade Apprenticeship	10	1	2.9
Level-3	231	28	16.8
Level-4+	474	58	40.2
Other	0	0	6.6
PNTD	15	–	–
Total	840	100.0	100.0
*Student status*			
Yes	227	27	5.7
No	599	73	94.3
PNTD	14	–	–
Total	840	100.0	100.0
Gender			
Female	553	66	50.6
Male	276	33	49.4
Other	8	1	–
PNTD	3	–	–
Total	840	100.0	100.0
*Employment status*			
Full-time	373	46	NA
Part-time	177	22	NA
Not in paid work	262	32	NA
PNTD	28	–	–
Total	840	100.0	NA

We conducted an online experiment to investigate the economic validity of the mere choice effect and test our main research hypothesis (H1). Participants were recruited via the research platform Prolific and sampled from a U.K. general population.^2^ Table 1 presents the descriptive statistics on the sample demographics. The sample shows the typical characteristics of an online panel (mean age was 33.1 years, SD $=$ 11.8).

**Fig. 1:**
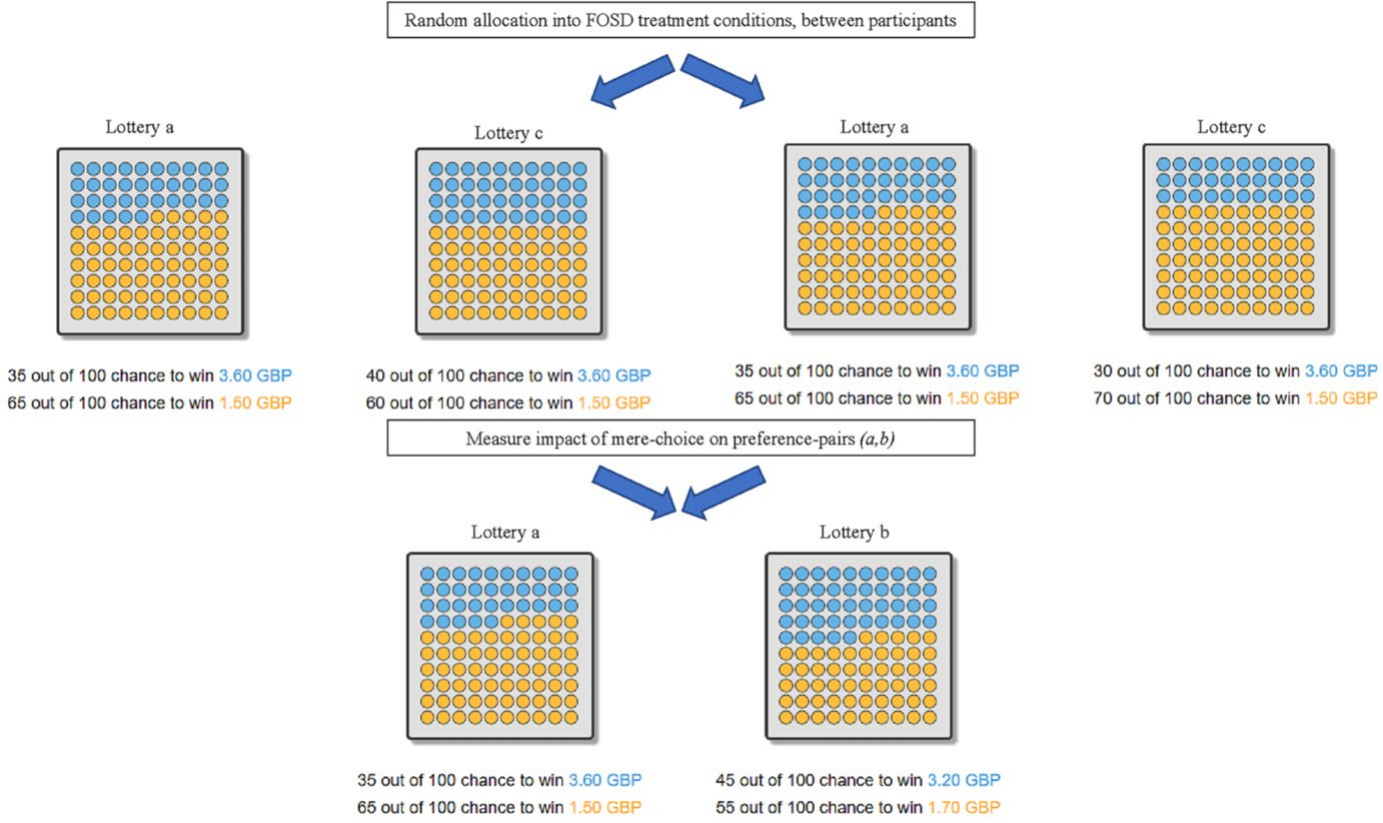
Schematic overview experimental design, including screenshots from actual decision screens. Lottery labels *a*, *b*, and *c* are for illustrative purposes only, and were not shown to participants

Each participant made 16 choices in total, each between two lotteries with two monetary outcomes and two probabilities. Eight choices were of the type (*a* vs. *c*), the remaining eight ones of the type (*a* vs. *b*). We implemented a standard between-participants design and randomized whether target lotteries *a* were inferior or superior to constructed lotteries *c* at the participant level. Lotteries were presented as icon arrays and we used a colored-balls-in-a-box framing (Garcia-Retamero and Galesic, 2010; Dambacher et al., 2016). All relevant design aspects of the presentation format were counterbalanced, e.g. colors, the position of the lotteries on screen, or the order of presentation within stages. Figure 1 summarizes the experimental design using sample screenshots and Table 2 shows the lotteries used in the experiment, each row representing one (*a*, *b*) lottery pair.

All participants first went through a standard attention screening, a typical procedure to reduce noise in online experiments (Oppenheimer et al., 2009).^3^ After passing the attention check, participants received detailed instructions on our lottery presentation format. They were then required to answer a small control quiz ensuring that they understood the lottery presentation format. After passing the quiz, each participant faced two decision stages, a mere-choice task in stage 1 and a preference-choice task in stage 2. In both choice tasks, participants were presented with pairs of lotteries, one pair at a time. They were instructed to choose the lottery they preferred in each pair.

The mere-choice task in stage 1 consisted of eight pairs of lotteries. Each mere-choice pair displayed one target lottery of type *a* (see Table 2) and a new lottery *c* constructed on the spot. Lotteries *c* were constructed to induce predetermined choice patterns and did not replicate any of the lotteries from Table 2. For the construction of *c*, we relied on transparent first-order stochastic dominance (FOSD). A lottery *a* first-order stochastically dominates another lottery *c* if for any monetary outcome *x*, *a* gives at least as high a probability of receiving at least *x* as does *c*, with strictly higher probability for some *x*. If a lottery first-order stochastically dominates another lottery, the former is objectively superior, independently of underlying risk preferences, as long as participants prefer larger amounts of money over smaller ones (the same remains true if decision makers are described correctly by cumulative prospect theory or rank-dependent utility instead of expected utility theory).

In the experiment, participants were randomly assigned to one of two possible treatments. In the CHOOSE treatment, all lotteries of type *a* dominated the corresponding *c*-type lotteries. Participants who obeyed FOSD thus ‘merely-chose’ *a*. In the REJECT treatment, the FOSD relationship was reversed so that participants obeying FOSD ‘merely-rejected’ *a*. To obtain transparent FOSD relationships, we changed one lottery attribute keeping the other one constant. For robustness reasons, we split the treatments into sub-treatments at the participant level, randomly determining whether probabilities were changed or whether monetary outcomes were changed (more details are provided below).

Our experimental set-up shares some elements with the standard asymmetric dominance / decoy effect design (Huber et al., 1982; Herne, 1999; Sürücü et al., 2019). That is, lottery *c* in the CHOOSE treatment is dominated by *a*, but not by *b*, making *a* more attractive than *b* as a result of the decoy effect. However, we remark that in our design the three lotteries are never presented on the same screen, but rather sequentially. Nevertheless, the decoy effect could still be present in our sequential presentation format, confounding the mere choice effect. We therefore implemented an additional sub-treatment in the CHOOSE condition to control for the decoy effect. When constructing *c*, we made lottery *c* so inferior monetary-wise that it was dominated by both *a* and *b*. We call such *c*-lotteries *junk* lotteries. With *junk* lotteries there is no asymmetric dominance present, and the decoy effect is shut down. To preserve symmetry between conditions, we used an analogous design for the REJECT condition. That is, *star* *c*-lotteries were made so attractive monetary-wise that they dominated both *a* and *b*.

The sub-treatments discussed above were counterbalanced between participants, yielding a 2 $\times$ 3 between-participants design with a total of six experimental conditions: CHOOSE with FOSD manipulation by probability, CHOOSE with FOSD manipulation by outcome, CHOOSE with *junk*-FOSD manipulation by outcome, REJECT with FOSD manipulation by probability, REJECT with FOSD manipulation by outcome, and REJECT with *star*-FOSD manipulation by outcome.^4^ Figure 1 includes a schematic overview of our FOSD construction for the probability domain. Randomization into treatments occurred after passing the control quiz.

The preference-choice task in stage 2 followed a setup analogous to the mere-choice task. It consisted of eight pairs of lotteries. Each preference pair presented one target lottery *a* and the corresponding lottery *b* given in the same row in Table 2. We thus had eight fixed preference pairs of the form (*a*, *b*) as given in Table 2.

**Table 2:** Lotteries used in experiment. Lotteries pay amount Outcome 1 with probability *p*, and Outcome 2 with probability $1-p$. All outcomes are in British Pounds (£), as Prolific is UK-based and compensates participants in £

Lotteries *a*						Lotteries *b*				
ID	*p*	Outcome 1	Outcome 2	EV		ID	*p*	Outcome 1	Outcome 2	EV
1	0.32	4.70	1.50	2.52		9	0.38	3.70	1.70	2.46
2	0.40	4.20	1.40	2.52		10	0.46	3.50	1.60	2.47
3	0.56	3.60	1.20	2.54		11	0.62	3.20	1.40	2.52
4	0.70	3.00	1.00	2.40		12	0.78	2.70	1.20	2.37
5	0.24	4.90	1.50	2.32		13	0.14	3.60	1.70	1.97
6	0.38	3.80	1.30	2.25		14	0.31	2.80	1.50	1.90
7	0.54	3.00	1.10	2.13		15	0.47	2.20	1.30	1.72
8	0.72	2.50	1.00	2.08		16	0.63	1.90	1.10	1.60

To incentivize decisions, we implemented a random lottery incentive system (Cubitt et al., 1998). A participant’s payment for the experiment was derived by selecting one of the sixteen lottery pairs from stage 1 and stage 2 at random. The participant then received the lottery she had chosen and that lottery was played out. This was done after all decision-relevant data was collected. On the basis of past experience with comparable experiments, the experiment was expected to last about 7 minutes and yield an average remuneration of £3.04.^5^ Actual average duration was 6 minutes and 34 seconds, and actual average remuneration was £3.09.

The lotteries in Table 2 were designed such that no FOSD relation obtains among any preference pair (*a*, *b*); lotteries of type *c* do not duplicate any of the existing lotteries *a* or *b*; all lotteries are non-degenerate, i.e., no certainty is involved; and the expected average payment of the experiment meets the current standards in experimental economics. The first four (*a*, *b*)-lottery pairs from Table 2 are hard / difficult decisions, because they involve a clear tradeoff, while the last four pairs are comparatively easier (see Sect. 4 for more details). The online appendix contains screenshots of all phases of the experiment.

### Measuring and testing the mere choice effect

In our design, the mere choice effect on future choices can be measured precisely. In preference-choices (*a*, *b*), merely-chosen target lotteries *a* should be chosen more often than merely-rejected target lotteries *a*. This effect is causal, because it was randomly determined whether the target lottery was merely-chosen or merely-rejected. Statistical significance is assessed via a Mann-Whitney-U (MWU) test, one-tailed as our hypothesis is directional. For the test, we count for each participant how often she chose lottery *a* in preference choices (*a*, *b*) (from 0 to 8). Let $x_{CHOOSE}$ and $x_{REJECT}$ denote one randomly drawn choice-count observation from each of the two treatments CHOOSE and REJECT, respectively. The MWU tests the following statistical hypotheses:^6^

**H0:** Probability$$[x_{CHOOSE} > x_{REJECT}]$$ $$\le$$ $$\frac{1}{2}$$

**Ha:** Probability$$[x_{CHOOSE} > x_{REJECT}]$$ > $$\frac{1}{2}$$.

We ﻿committed to conclude to have found supportive evidence of a mere choice effect if and only if the MWU test was significant at the 5% level.

### Power calculations

We expected a small effect size and hence set $d=0.2$ for power calculations (Cohen, 1988, 1992); for example, the related literature on choice-induced preference change in psychology reports an average effect size of $d = 0.26$ (Izuma and Murayama, 2013). Setting $\alpha = 0.05$, $1-\beta =0.8$, and $d=0.2$, the *a priori* required sample size for a one-tailed MWU test is 650 participants, equally split between treatments. Hence, the research question is best tackled by a large-sample but rather short experiment, and hence an online platform is ideal.

### FOSD and exclusion criteria

To ensure that the FOSD manipulation induced behavior as expected, independently of other factors, we aimed to maximize the transparency of FOSD relationships. We therefore changed one lottery attribute keeping the other one constant. In the probability domain, FOSD relationships were established by adding or subtracting five percentage points in probabilities for the higher outcome. In the outcome domain, we added or subtracted 20 pence to or from the high outcome. To create *junk* (*star*) type-*c* lotteries, the highest (lowest) outcome in *c* was set equal to the lowest (highest) outcome in both *a* and *b* lotteries. For example, let (*x*, *p*; *y*) denote a lottery that pays *x* with probability *p* and *y* with the complementary probability $1-p$. Let the target lottery be $a = (12, 0.25; 2)$ and $b= (8, 0.5; 3)$. Suppose we wish to construct a lottery *c* so that *a* is to be chosen in the pair (*a*, *c*). In the probability domain, we would construct $c = (12, 0.20; 2)$. In the outcome domain, we would either set $c = (11.8, 0.25; 2)$ or *junk*-$c = (2, 0.25; 1)$. In the former case, *c* pays the same amounts as *a*, but entails a lower probability to win the higher amount. In the latter case, *c* simply pays less money, but the probabilities are the same as in *a*. If behavior follows FOSD, participants are expected to choose *a* in all three cases.

However, it is possible that some participants violate FOSD, e.g. due to lack of attention. We committed to excluding participants who violate FOSD in at least one of the eight mere-choice pairs from the analysis. Alós-Ferrer et al. (2016a) conducted a laboratory experiment with a standard student population. The authors included FOSD-choice pairs similar to ours as a basic rationality check in their experiment, which was designed to test an unrelated phenomenon (the preference reversal phenomenon). The authors report extremely low FOSD violation rates (around 2%). As in Alós-Ferrer et al. (2016a), we use incentivized choice, and our lottery presentation format relies on icon-arrays which communicate risk understandably to lay audiences (Garcia-Retamero and Galesic, 2010; Dambacher et al., 2016). Taking into account the noisier online environment, we therefore expect FOSD violations rates of 5%. We conservatively set to obtain the required number of 650 observations after a 5% of exclusions, leading to a required number of participants of 682, which we conservatively rounded up to 720. We additionally invited 120 participants to complete a baseline measurement treatment for choice frequencies in (*a*, *b*)-pairs. Further information regarding this baseline treatment is provided later on. In total, we thus recruited 840 participants. We committed to performing our main tests with all remaining participants after excluding those who violated FOSD at least once.

This exclusion is based on objective criteria and does not compromise a causal interpretation of our results. First, the two treatments CHOOSE and REJECT only differ with respect to whether *c* is objectively better or worse than *a*. Otherwise, they are identical. Participants are blind with regard to the identities of the lotteries, they do not know which lottery is of type *a*, *c*, or *b*. Hence, FOSD violations are pure noise and we do not expect FOSD violation rates to vary across treatments.^7^ Second, mere choices do not carry any *a priori* relevant information for preference pairs (*a*, *b*). Hence, our exclusion criterion does not condition on any relevant information with regard to the measurement of the mere choice effect. Admittedly, one can take the position that excluding participants limits the generalizability of our conclusions, and that all results stated hold only for the subset of participants who obey FOSD in the mere-choice task (or actually pay attention to the task). However, we expected this subset to be large.

## Results

### FOSD violations

In total we recruited 720 participants for the CHOOSE and REJECT treatments. We had to exclude 2 participants as their choices were not recorded properly. We thus observed 5,744 (718 participants $\times$ 8 decisions) decisions in which one lottery dominated the other one in the FOSD sense in stage 1. Only a small fraction of these decisions violated FOSD in CHOOSE and REJECT, respectively 134 (4.7%) and 154 (5.4%). We further split up the data across manipulation domains, i.e., outcomes, outcomes star/junk and probabilities. FOSD violation rates were again low, respectively 118 (6.1%), 24 (1.3%) and 146 (7.6%). Following our plan, we excluded 132 of the 718 participants since they violated FOSD at least once, leaving us with 586 participants for our main analysis, 298 in CHOOSE and 288 in REJECT. Unless otherwise stated, our analysis is based on the sample of 586 participants who obeyed FOSD in all of their choices in stage 1.

### Mere choice effect

**Fig. 2:**
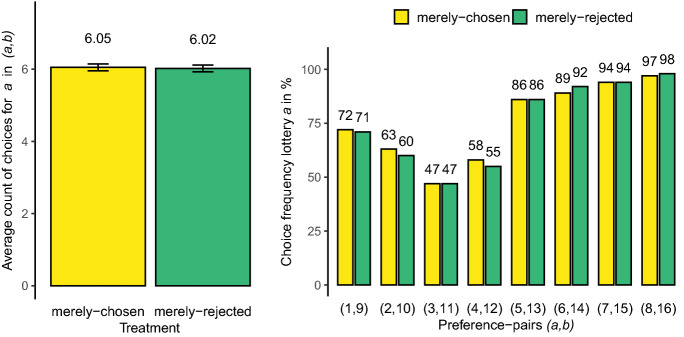
Left-hand panel: Average count of choices for lottery *a* in $\mathbf{a} ,\mathbf{b}$ across mere-choice treatments. Right-hand panel: Choice frequencies for lottery *a* across preference pairs $\mathbf{a} ,\mathbf{b}$ and mere-choice treatments

The left-hand side of Fig. 2 plots the average number of times that lottery *a* was chosen across participants (0 to 8) for the CHOOSE and REJECT treatments. With 6.05 in the CHOOSE treatment vs. 6.02 in the REJECT treatment, the participant-average count of choices for *a* in (*a*, *b*) in treatment CHOOSE was almost identical to the one in treatment REJECT (medians were 6 and 6, respectively). These observations are corroborated by a one-sided MWU test on differences in the distribution of *a*-choices between treatments ($z=$ 0.33, $p=$ 0.37), see Sect. 3.3. Uninformative mere choices did not significantly increase the choice frequencies of merely-chosen lotteries. For illustrative purposes, the right-hand side of Fig. 2 also plots the choice frequencies for lottery *a* in preference pairs (*a*, *b*) for the CHOOSE and REJECT treatments, for each of the eight preference pairs (*a*, *b*) separately. Against our main hypothesis, we observe that merely-chosen lotteries *a* were chosen at the same rates as comparable, but merely-rejected lotteries *a* in all preference pairs.

### Robustness analysis

We ran panel probit regressions with participant random-effects to confirm our main analysis on the mere choice effect. Our sample comprises all decisions made in stage 2 of the experiment excluding participants who violated FOSD at least once in stage 1. Our dependent variable is the *Choice* dummy, taking the value 1 if a participant chose *a* in (*a*, *b*). Reported are average marginal effects with cluster-robust standard errors in parentheses (that is, treating each individual as a cluster). The corresponding results are presented in Table 3, Models (1) and (2).

**Table 3:** Panel probit regressions on Choice dummy (choose *a* in (*a*, *b*)) with participant random-effects in Models (1) to (4). Reported are average marginal effects with cluster-robust standard errors in parentheses. Model (5) reports the results of a panel linear probability model with participant random-effects and cluster-robust standard errors in parentheses

Dependent variable	Choice dummy, choose *a* in (*a*, *b*)				
Model	(1)	(2)	(3)	(4)	(5)
Merely-Chosen	0.004	0.013	− 0.005		− 0.001
	(0.017)	(0.016)	(0.015)		(0.015)
CHOOSE				0.012	
				(0.020)	
CHOOSE-JUNK				0.013	
				(0.025)	
REJECT-STAR				0.000	
				(0.025)	
Hard					− 0.266**
					(0.024)
Merely-Chosen $\times$ Hard					0.027
					(0.027)
Position screen: Right		0.036***	0.037***	0.036***	0.036***
		(0.011)	(0.010)	(0.011)	(0.011)
Winning color: Orange		0.020	0.022	0.020	0.019
		(0.016)	(0.015)	(0.016)	(0.016)
Female		− 0.053***	− 0.059***	− 0.053***	− 0.056***
		(0.017)	(0.016)	(0.017)	(0.017)
Age		− 0.002*	− 0.003***	− 0.002*	− 0.002**
		(0.001)	(0.001)	(0.001)	(0.001)
Student status (baseline NO))					
YES		− 0.050*	− 0.44*	− 0.050*	− 0.053*
		(0.027)	(0.024)	(0.028)	(0.028)
PNTD		0.048	− 0.001	− 0.001	0.040
		(0.056)	(0.060)	(0.056)	(0.053)
*Education level (baseline Level-1)*					
No qualification		0.061	0.007	0.061	0.055
		(0.112)	(0.088)	(0.112)	(0.104)
Level-2		0.085	0.007	0.085	0.084
		(0.056)	(0.052)	(0.056)	(0.057)
Trade apprenticeship		0.081	− 0.063	0.081	0.067
		(0.112)	(0.116)	(0.113)	(0.117)
Level-3		0.040	− 0.008	0.041	0.038
		(0.051)	(0.046)	(0.052)	(0.052)
Level-4+		0.102**	0.040	0.102**	0.101**
		(0.047)	(0.045)	(0.049)	(0.049)
PNTD		− 0.027	− 0.060	− 0.027	− 0.025
		(0.080)	(0.068)	(0.080)	(0.075)
Employment status (baseline Full-time))					
Part-time		0.048**	0.047**	0.048**	0.046
		(0.024)	(0.022)	(0.024)	(0.024)
Not in paid work		0.050**	0.046**	0.050**	0.055**
		(0.023)	(0.022)	(0.023)	(0.025)
PNTD		0.001	0.009	0.008	0.008
		(0.044)	(0.041)	(0.044)	(0.045)
Income (baseline < 19,000 ))					
19,000 to 31,999		− 0.002	0.012	− 0.002	− 0.002
		(0.026)	(0.025)	(0.026)	(0.027)
32,000 to 47,999		− 0.007	0.017	− 0.007	− 0.004
		(0.025)	(0.024)	(0.025)	(0.026)
48,000 to 63,999		− 0.031	0.002	− 0.032	− 0.029
		(0.030)	(0.028)	(0.030)	(0.030)
64,000 or more		0.057**	0.065**	0.057**	0.061**
		(0.029)	(0.028)	(0.029)	(0.030)
PNTD		0.010	0.002	0.010	0.014
		(0.035)	(0.033)	(0.035)	(0.035)
Constant					0.917***
					(0.069)
Number of participants	586	586	718	586	586
Number of observations	4688	4688	5744	4688	4688
FOSD violations	No	No	Yes	No	No
Period fixed-effects	No	Yes	Yes	Yes	Yes
Lottery fixed-effects	No	Yes	Yes	Yes	Yes

Significance codes: *$$p<.10$$**$$p<.05$$ ***$$p<.01$$

Our regression analysis confirms our main findings from Sect. 4.2. The Merely-Chosen dummy in Model (1), taking value 1 if lottery *a* was merely-chosen, is insignificant. This dummy captures the grand difference between merely-chosen and merely-rejected *a* lotteries in our experiment. These results are robust with regard to preference-pair-specific features, period effects, demographic controls, and presentation controls, see Model (2). Demographic control variables were included for robustness purposes, but we had no specific hypotheses about them. We observe that women, students, and older participants tended to choose lottery *a* less often. Similarly, highly educated participants, participants not in full-time work, and participants belonging to the highest household income level tended to choose lottery *a* more frequently. We also found that lottery *a* was chosen more frequently when positioned on the right-hand side of the screen. Finally, Model (3) is equivalent to Model (2) in terms of specification, but includes participants who violate FOSD, with FOSD violations coded as the actual choice made in the mere-choice pair. We expected Model (3) to yield similar results as Model (2) for two reasons. First, we expected FOSD violation rates to be low, so their impact should be small. Second, the underlying theories on choice-induced-preference changes do not distinguish between correct and erroneous decisions. Incorrectly chosen (dominated) lotteries *a* should trigger the same preference change effects as correctly chosen (dominating) lotteries *a*. Model (3) broadly confirms our previous analysis and we do not find any trace of the mere-choice effect.

Model (4) controls for the decoy effect as explained in Sect. 3 of the paper. This models drops the Merely-Chosen dummy and replaces it with dummy variables representing different treatments. The comparison treatment is given by the non-*star* REJECT treatments. The CHOOSE dummy represents the non-*junk* CHOOSE treatments. The CHOOSE-JUNK and REJECT-STAR dummies take the value 1 if lottery *c* was a *junk*-type and *star*-type lottery, respectively. As can be seen in Table 3, the CHOOSE-JUNK and CHOOSE coefficients are insignificant. The mere choice effect was, hence, absent in our data independently of how we constructed lottery *c*. In the CHOOSE treatments, *c* was dominated by *a*, but not by *b*. In CHOOSE-JUNK, *c* was dominated by both *a* and *b*. There was, hence, no asymmetric dominance present in the latter observations and the decoy effect was shut down. Comparing the CHOOSE-JUNK and CHOOSE coefficients with a post-hoc hypothesis test, we find this difference to be insignificant. Absence of evidence for the decoy effect is likely due to our sequential presentation format. That is, the decoy effect emerges only when all three relevant choice options are presented simultaneously. We also observe that the REJECT-STAR dummy is not significant. With non-*star* REJECT treatments asymmetric dominance should impact the desirability of lottery *c*; *c* dominates *a*, but not *b*. Hence, no difference between the REJECT treatments was to be expected, since all choices in stage 2 were among (*a*, *b*), not involving *c*.

Model (5) controls for the difficulty of the decision. It is known from the literature that hard / difficult decisions, i.e., being close to indifference, typically generate more variability in choice, higher preference reversal rates, or stronger decoy effects (see, e.g., Moffatt, 2005; Alós-Ferrer et al., 2016a; Agranov and Ortoleva, 2017; Alós-Ferrer, 2018; Alós-Ferrer and Garagnani, 2018; Sürücü et al., 2019; Alós-Ferrer and Garagnani, 2021). It is plausible to assume that the preference increase resulting from mere choices has a natural, upper bound. In this case, the mere choice effect would be strongest if the decision maker is close to indifference in (*a*, *b*) decisions. That is, if *ex ante* *a* and *b* are similar in magnitude in terms of expected utilities, mere-choice-induced preference changes are more likely to tilt the decision in favor of target lottery *a*. We can classify the first four of our (*a*, *b*)-lottery pairs from Table 2 as hard / difficult decisions, because they involve a clear tradeoff. Lottery *a* has a higher expected value than *b*, but is more risky (the lower outcome has a larger probability). Analogously, we can classify the last four (*a*, *b*)-lottery pairs as easy decisions. There is no tradeoff in these pairs and the higher expected value lotteries *a* are relatively safe.^8^ In easy decisions, lottery *a* is the favorite and we expect that *a* is chosen more frequently than *b* (>50%). In hard decisions we expect participants to be closer to indifference, with choice frequencies also closer to 50:50. To control for the difficulty of decisions, we have added a dummy variable in Model (5) that captures whether or not (*a*, *b*) were hard/difficult decisions. We have also included an interaction term between the Merely-Chosen dummy and the the HARD dummy to investigate if the mere choice effect depends on decision difficulty. We opted for a linear probability approach in Model (5), because it is impossible to estimate the marginal effect of an interaction term using probit. As expected, the HARD dummy was significant and negative. On average, the choice frequency for *a* was 26.6 percentage points lower in hard decisions than in easy ones, see also the right-hand panel in Fig. 2. We also observe that the interaction term is insignificant, providing evidence that the mere-choice effect was also absent in difficult decisions.

As a last robustness check, we have run additional linear probabilities models which we report in the Online Appendix. The aim of these models was to investigate if the manipulation domain (probabilities vs outcomes) to obtain FOSD relationships impacted the mere choice effect. The models included an interaction term between the Merely-Chosen dummy and a dummy capturing the probability domain. We find no evidence for such an effect and the estimated interaction coefficients were not significant.

Finally, we would like to note that, in general, an observed mere choice effect could have been a manifestation of the mere exposure effect, see also Sect. 2. The mere exposure effect is a general tendency in decision makers to develop a ‘preference’ for alternatives merely because they have been exposed to them (Zajonc, 1968; Bornstein, 1989; Monahan et al., 2000; Zajonc, 2001). To test for the mere exposure effect, we ran an additional baseline treatment with 120 participants. In this baseline treatment, participants made FOSD choices in stage 1 unrelated to the pairs (*a*, *b*). Stage 2 was identical to REJECT and CHOOSE. When making choices between (*a*, *b*) in stage 2, no participant was, hence, previously exposed to the *a*-type lotteries in the baseline treatment. In comparison to the baseline treatment, the mere exposure effect should increase the choice frequency for *a* in both CHOOSE and REJECT in stage 2. To facilitate comparison between treatments, we followed an analogous procedure as for our main tests and eliminated 25 participants who violated FOSD at least once with their stage-1 choices in the baseline treatment (the FOSD violation rate was 6.1% in this treatment). A Kruskal-Wallis test did not reveal any significant differences in the number of choices for *a* in (*a*, *b*) per participant between CHOOSE, REJECT, and the baseline treatment ($p = 0.78$). We recorded on average 6.05, 6.02, and 6.06 choices for *a*-type lotteries in stage 2 in CHOOSE, REJECT, and baseline, respectively. That is, we find no trace of the mere exposure effect in our data.

## Conclusion

Using a novel, parsimonious experimental design, we have presented the first conclusive evidence on the economic validity of the mere-choice-induced preference change phenomenon. We do not find any evidence which could be interpreted as mere-choice-induced preference change. Of course, absence of evidence is not evidence of absence, but, given the power analysis underlying our analysis, the simplest explanation for our results at this point is that mere-choice-induced preference change in economic domains does not exist or is of a negligible magnitude.

From predicting consumer behavior to cost-benefit analyses of medical treatments to welfare comparisons of alternative market institutions, many applications of standard theories of decision making under risk are built on the possibility to organize observed choices through underlying stable preferences. We have shown that the latter view seems appropriate with regard to mere-choice-induced preference changes.

Of course, as with any other experiment finding a null effect, it might still be the case that the alleged effect exists under some additional condition not fulfilled in our design. For instance, we have manipulated choice in lottery pairs by previous choices involving the riskier of the two lotteries in the pair, in the sense that the two monetary outcomes of that lottery are slightly more extreme than the ones of the alternative. However, as the mere-choice effect is understood in the literature, it should have been effective in our experiment, and additional conditions would come on top of received descriptions of the alleged effect.

We should also remark that we have studied the pure effect of uninformative choice on preference. A related stream of literature in psychology, which regrettably used a flawed design (see Alós-Ferrer and Shi, 2015, for details), can be seen as incorporating some form of tradeoff in choice. If tradeoffs are a necessary precondition for the phenomenon to emerge then appropriate experimental designs will have to be developed, with an eye on separating this potential source from the pure effect of choice. At this point, however, we can conclude that the phenomenon of mere-choice-induced preference change is weak or nonexistent and, therefore, probably not very relevant in economically-relevant domains.

## Supplementary Information

Below is the link to the electronic supplementary material.

Supplementary file1 (PDF 595 kb)

## Footnotes

1 The described self-inference process is related to a recent stream of literature on motivated reasoning in economics. Motivated reasoning can be a forceful driver of people’s shifts in beliefs and attitudes. Bénabou and Tirole (2016) provides an overview. The mere choice phenomenon suggests that (pressumably unconscious) motivated reasoning may also apply to the domain of preferences.

2 Prolific is a U.K.-based, well-established research platform and is increasingly gaining popularity in economics (Palan and Schitter, 2018; Kong et al., 2020).

3 We used an instructional attention check, see Figure A.2 in the online appendix. Participants were instructed to ignore the question text and to simply answer the question in a specific way by entering the word ‘clear’ into a text field.

4 This design allows us to both establish whether the decoy effect is present in sequential presentation formats and to deal with this potential confound effectively. The *junk* / *star* construction process was implemented for outcome manipulations only for a simple reason. One aim of our paper was to investigate whether the manipulation domain impacts the mere choice effect. This requires changes in the FOSD construction process (*ceteris paribus*): keeping probabilities constant and changing outcomes, or keeping outcomes constant and changing probabilities. It is not possible to construct *junk* and *star* lotteries *c* by changing probabilities alone.

5 In accordance with the recommendations set by the Prolific team, participants are paid a flat completion fee of £0.60. Assuming that all choices comply with FOSD, the expected value of our random lottery incentive system is £2.44. Hence, expected earnings are £0.60 + £2.44 = £3.04. This is about three times as high as the current highest minimum wage rate in the UK.

6 The stated null hypothesis is also known as stochastic inequality. The interpretation of stochastic inequality is straightforward: one sample stochastically tends to generate higher values than the other sample. Hereby, we assume equal-variances across samples, which is standard in experimental economics. A thorough discussion of the MWU test and why stochastic inequality is the ’correct’ null hypothesis is provided in Divine et al. (2018).

7 In theory, one could expect different FOSD violations rates across treatments, because under concave expected utility $u(x+0.2)-u(x) < u(x)-u(x-0.2)$. The left-hand side of the inequality represents the difference in expected utilities between a target lottery *a* and lottery *c* in the CHOICE treatment with FOSD manipulations in the outcome domain. The right-hand side represents the same difference in the corresponding REJECT treatment. If the decision maker is guided by EU with Fechner-type errors, FOSD violations in the former treatment are more likely than in the latter one. However, the empirical magnitude of this postulated effect is unlikely to be consequential. First, utility is typically very close to linear in the amounts typically used in experimental economics (Rabin, 2000). Second, as argued in Loomes and Sugden (1998) and Loomes et al. (2002), calibrated Fechner-type errors predict too many violations of transparent FOSD, compared to actual rates.

8 Garagnani (2020) recently estimated the CRRA risk parameter on a large Prolific U.K. population using standard choice lists and obtained a median $r=0.411$. Differences in expected utilities using this estimate are very close to zero for hard decision pairs, but are much larger for easier ones. We report these estimates in the online appendix.
