# Supplementary material for: Does choice change preferences? An incentivized test of the mere choice effect
Source: Exp Econ. 2021 Aug 15;26(3):499–521. doi: 10.1007/s10683-021-09728-5 (PMC10319671; doi:10.1007/s10683-021-09728-5)
Supplement: Supplementary file 1 — Supplementary file1 (PDF 595 kb) [file 10683_2021_9728_MOESM1_ESM.pdf]

Does Choice Change Preferences?
An Incentivized Test of the Mere Choice Effect

Carlos Alós-Ferrer and Georg D. Granic

ONLINE APPENDIX

A Additional results

We analyze here whether the mere choice effect differs between FOSD manipulation domains. We expected no differences between the manipulations domains, which were included for robustness purposes. To this end, we run panel linear probability models using the same specifications as in Models (2) and (3) of Table 3 in the main text. We included an interaction term between the mere-choice treatment dummy and the domain manipulation dummy. The results are presented in Model (1) and (2) of Table A.1. We opted for a linear probability model, because it is impossible to estimate the marginal effect of an interaction term in non-linear models like probit. We found no influence of the manipulation domain on the mere choice effect.

Table A.1 Panel GLS regressions on Choice dummy (choose a in (a,b)) with participant random-effects. Presented are the coefficient estimates with cluster robust standard errors in parentheses. Significance codes: * $p < .10$ ** $p < .05$ *** $p < .01$.

Dependent variable	Choice dummy, choose a in (a,b)	
Model	(1)	(2)
Merely-Chosen	0.000	0.008
	(0.021)	(0.019)
FOSD Manipulation Domain: Probabilities	-0.030	-0.027
	(0.024)	(0.022)
Merely-Chosen ×	0.009	-0.30
FOSD Manipulation Domain: Probabilities	(0.034)	(0.022)
Position screen: Right	0.036***	0.039***
	(0.011)	(0.011)
Winning color: Orange	0.017	0.021
	(0.017)	(0.015)
Female		-0.062***
		(0.016)
Age		-0.003***
		(0.001)
Student status (baseline NO)		
YES		-0.045*
		(0.024)
PNTD		0.000
		(0.053)
Education level (baseline Level-1)		
No qualification		0.007
		(0.085)
Level-2		0.002
		(0.053)
Trade apprenticeship		-0.084
		(0.117)
Level-3		-0.008
		(0.047)
Level-4+		0.037
		(0.045)
PNTD		-0.077
		(0.066)
Employment status (baseline Full-time)		
Part-time		0.045**
		(0.022)
Not in paid work		0.051**
		(0.023)
PNTD		0.015
		(0.040)
Income (baseline < £19,000)		
£19,000 to £31,999		0.016
		(0.025)
£32,000 to £47,999		0.020
		(0.024)
£48,000 to £63,999		0.010
		(0.028)
£64,000 or more		0.069**
		(0.029)
PNTD		0.004
		(0.033)
Constant	0.665***	0.736***
	(0.070)	(0.063)
Number of participants	586	718
Number of observations	4,688	5,744
FOSD violations	No	Yes
Period fixed-effects	Yes	Yes
Lottery fixed-effects	Yes	Yes
Interaction treatments	Yes	Yes

Table A.2 $EV()$: expected value of lotteries used in experiment. $EU()$: expected utility of lotteries used in experiment. $EU()$ calculation is based on CRRA utility with $r = 0.411$.

Lotteries a			Lotteries b				
ID	$EV(a)$	$EU(a)$	ID	$EV(b)$	$EU(b)$	DiffEV	DiffEU
1	2.524	1.660	9	2.460	1.669	0.064	-0.009
2	2.520	1.663	10	2.474	1.674	0.046	-0.011
3	2.544	1.681	11	2.516	1.693	0.028	-0.013
4	2.400	1.637	12	2.370	1.645	0.030	-0.008
5	2.316	1.577	13	1.966	1.473	0.350	0.104
6	2.250	1.558	14	1.903	1.445	0.347	0.113
7	2.126	1.518	15	1.723	1.366	0.403	0.152
8	2.080	1.515	16	1.604	1.311	0.476	0.204

B Experimental materials

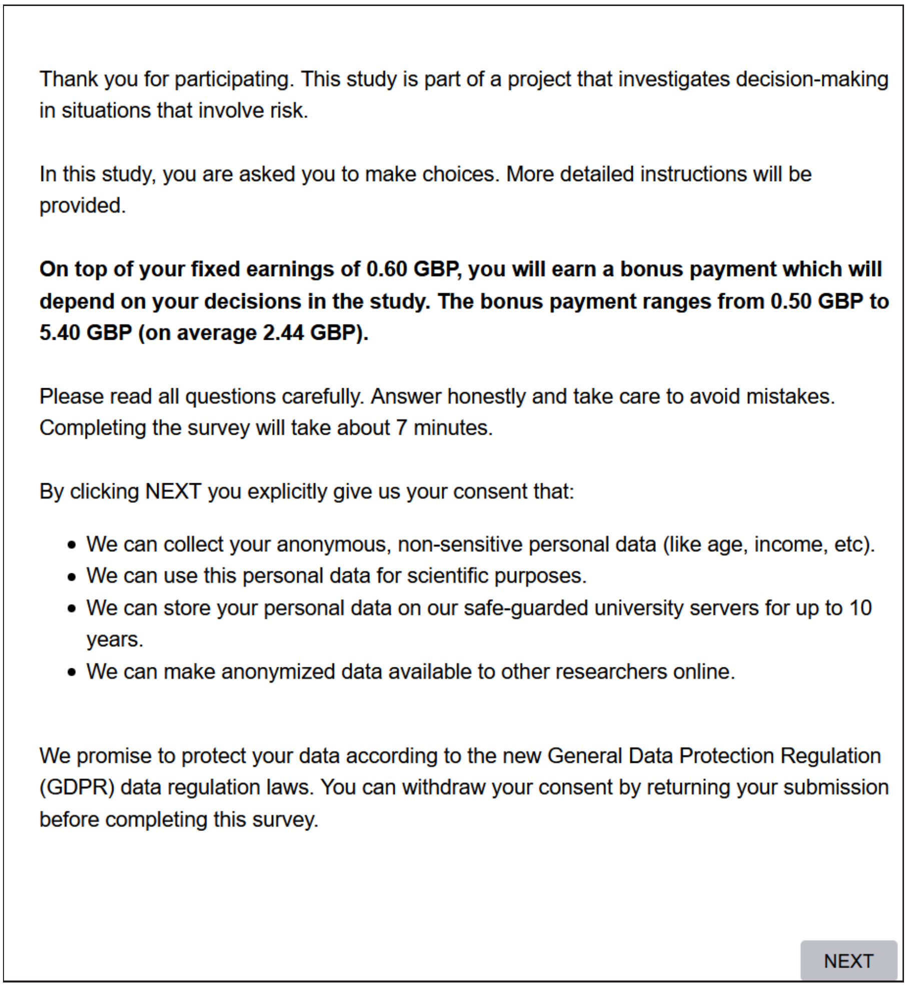

Thank you for participating. This study is part of a project that investigates decision-making in situations that involve risk.

In this study, you are asked you to make choices. More detailed instructions will be provided.

On top of your fixed earnings of 0.60 GBP, you will earn a bonus payment which will depend on your decisions in the study. The bonus payment ranges from 0.50 GBP to 5.40 GBP (on average 2.44 GBP).

Please read all questions carefully. Answer honestly and take care to avoid mistakes. Completing the survey will take about 7 minutes.

By clicking NEXT you explicitly give us your consent that:

- We can collect your anonymous, non-sensitive personal data (like age, income, etc).
- We can use this personal data for scientific purposes.
- We can store your personal data on our safe-guarded university servers for up to 10 years.
- We can make anonymized data available to other researchers online.

We promise to protect your data according to the new General Data Protection Regulation (GDPR) data regulation laws. You can withdraw your consent by returning your submission before completing this survey.

NEXT

Fig. A.1 Screen 1 online experiment: General introduction and consent.

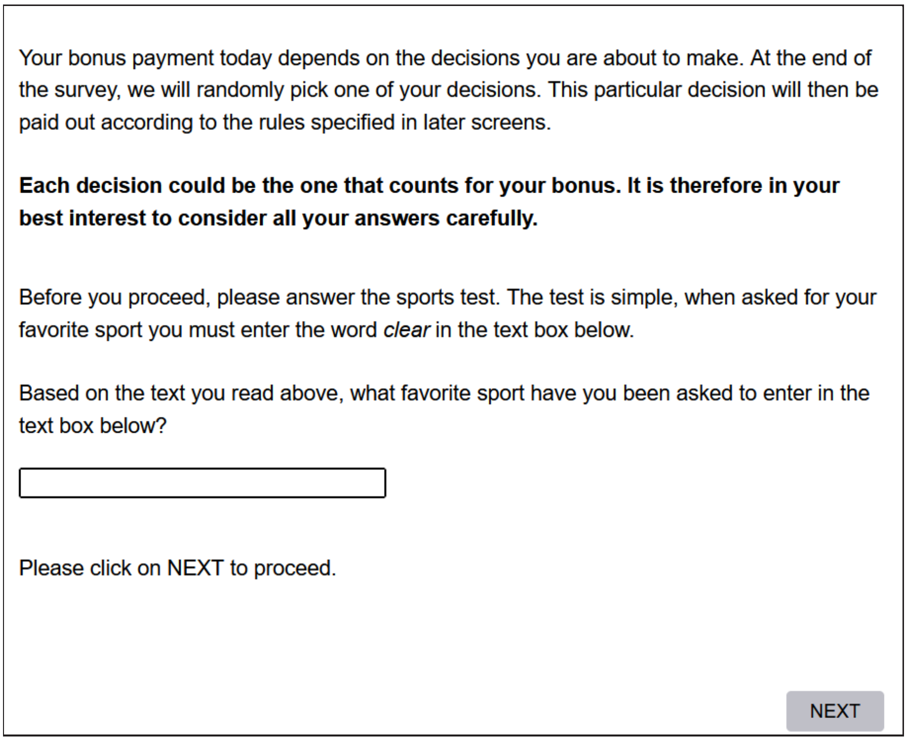

Your bonus payment today depends on the decisions you are about to make. At the end of the survey, we will randomly pick one of your decisions. This particular decision will then be paid out according to the rules specified in later screens.

Each decision could be the one that counts for your bonus. It is therefore in your best interest to consider all your answers carefully.

Before you proceed, please answer the sports test. The test is simple, when asked for your favorite sport you must enter the word *clear* in the text box below.

Based on the text you read above, what favorite sport have you been asked to enter in the text box below?

Please click on NEXT to proceed.

NEXT

Fig. A.2 Screen 2 online experiment: Random lottery incentives and attention check.

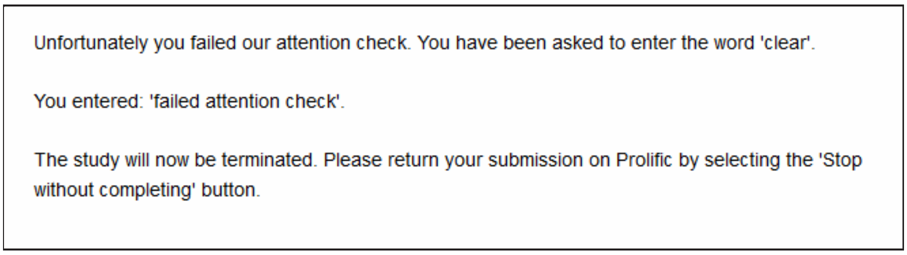

Unfortunately you failed our attention check. You have been asked to enter the word 'clear'.

You entered: 'failed attention check'.

The study will now be terminated. Please return your submission on Prolific by selecting the 'Stop without completing' button.

Fig. A.3 Screen 2a online experiment: Failed attention check.

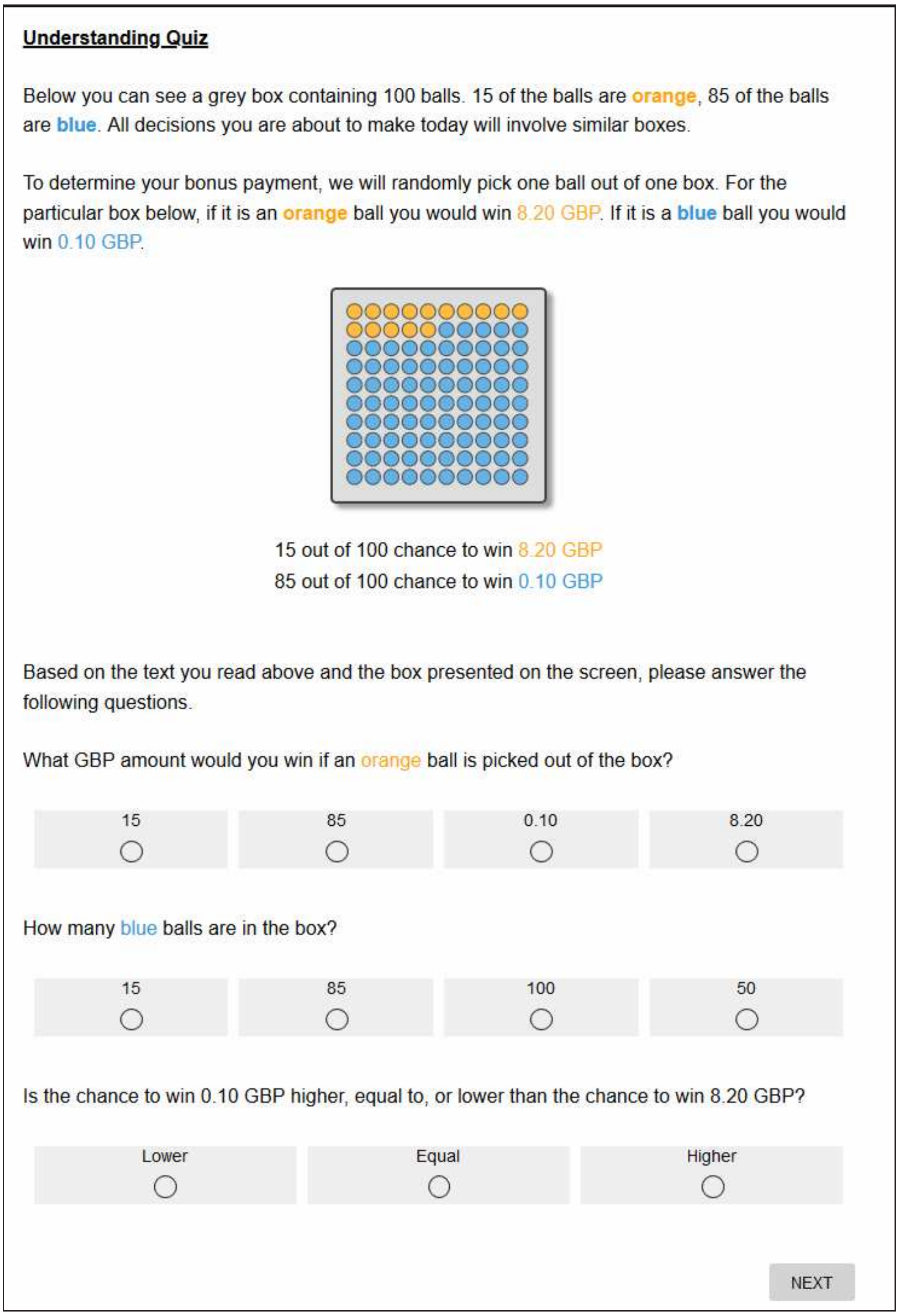

Understanding Quiz

Below you can see a grey box containing 100 balls. 15 of the balls are **orange**, 85 of the balls are **blue**. All decisions you are about to make today will involve similar boxes.

To determine your bonus payment, we will randomly pick one ball out of one box. For the particular box below, if it is an **orange** ball you would win 8.20 GBP. If it is a **blue** ball you would win 0.10 GBP.

15 out of 100 chance to win 8.20 GBP
85 out of 100 chance to win 0.10 GBP

Based on the text you read above and the box presented on the screen, please answer the following questions.

What GBP amount would you win if an orange ball is picked out of the box?

15	85	0.10	8.20
○	○	○	○

How many blue balls are in the box?

15	85	100	50
○	○	○	○

Is the chance to win 0.10 GBP higher, equal to, or lower than the chance to win 8.20 GBP?

Lower	Equal	Higher
○	○	○

NEXT

Fig. A.4 Screen 3 online experiment: Understanding quiz.

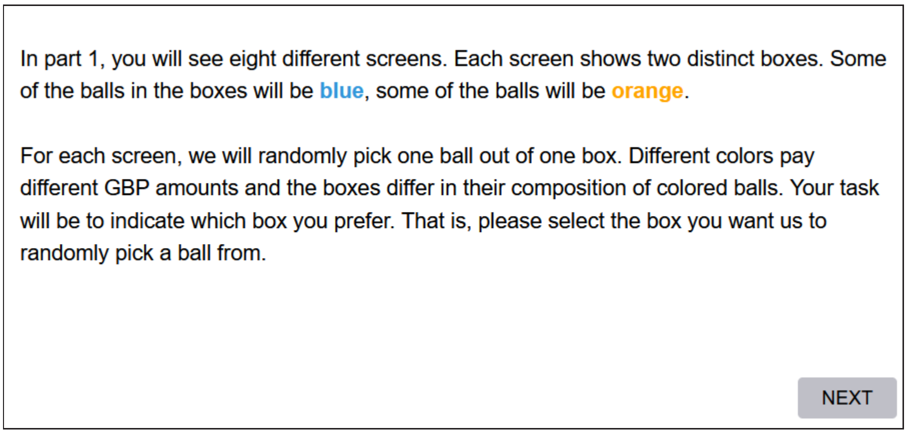

Fig. A.5 Screen 4 online experiment: Mere-choice task introduction.

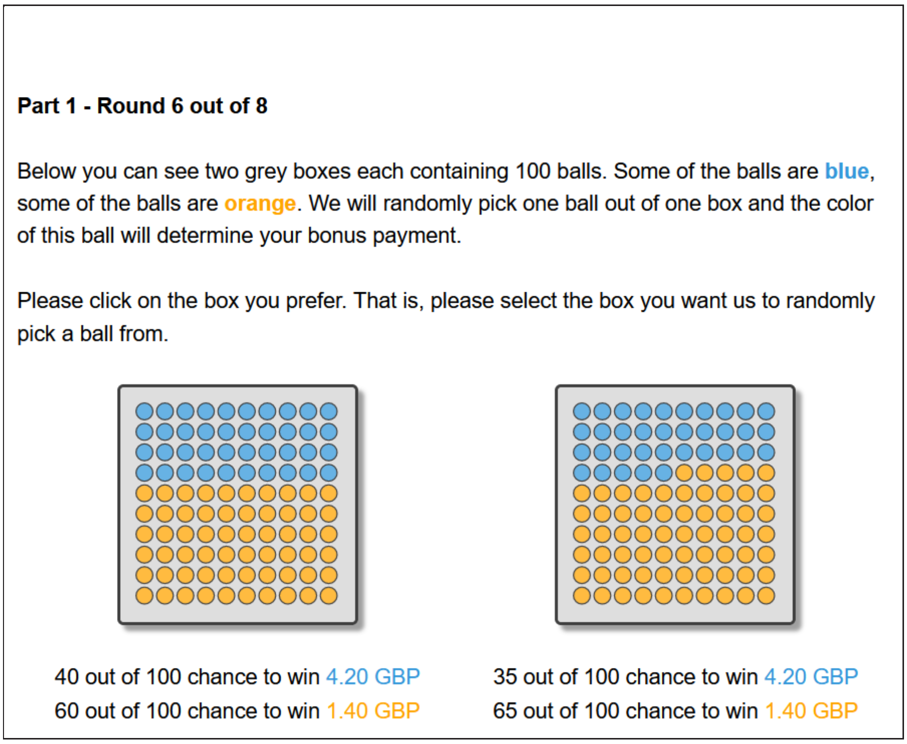

Fig. A.6 Screen 5 online experiment: Mere-choice task, CHOOSE treatment, lottery a on left-hand side of screen ($ID = 2$), FOSD on probabilities.

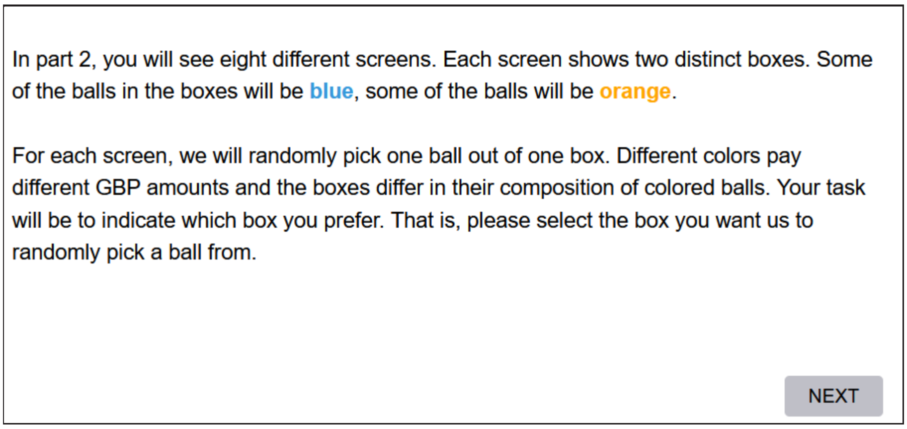

Fig. A.7 Screen 6 online experiment: Preference-choice task introduction.

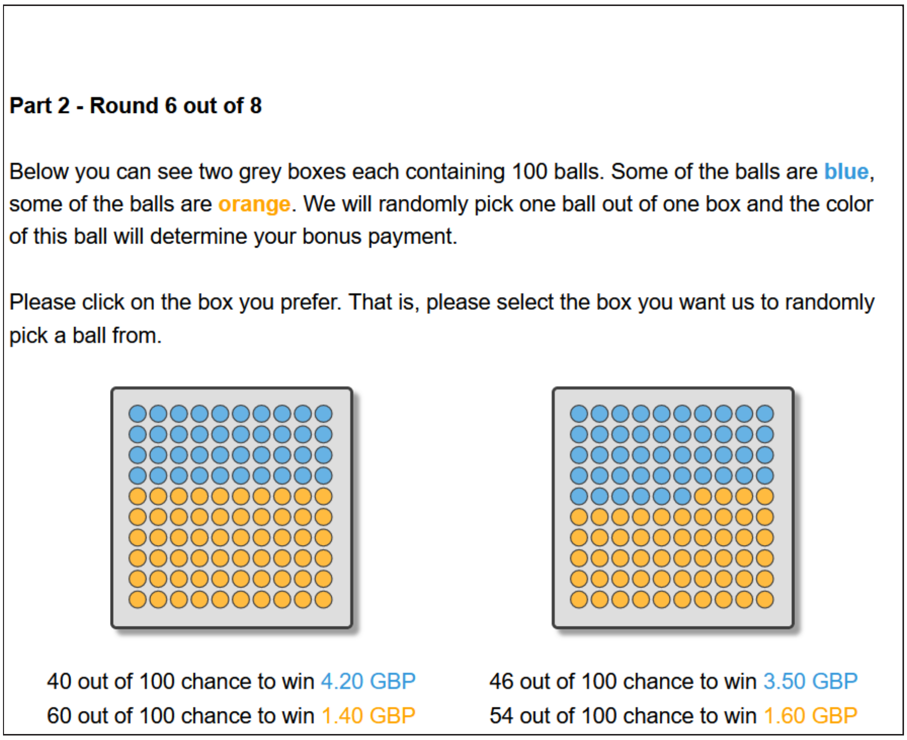

Fig. A.8 Screen 7 online experiment, preference pair $(2,10)$, lottery a on left-hand side of screen.

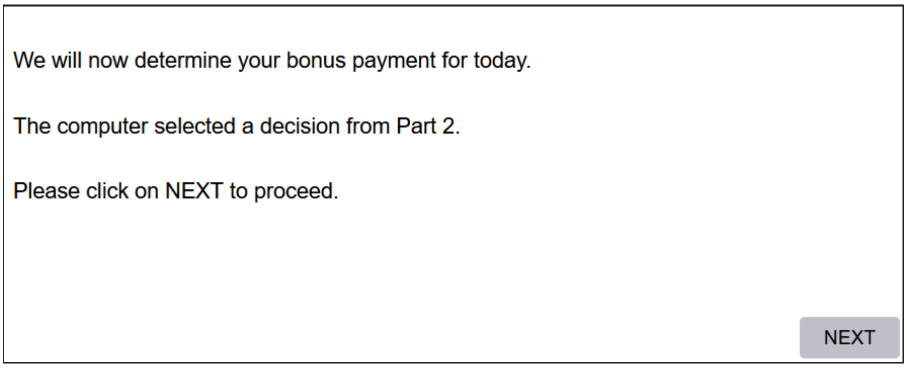

Fig. A.9 Screen 8 online experiment: Payment introduction.

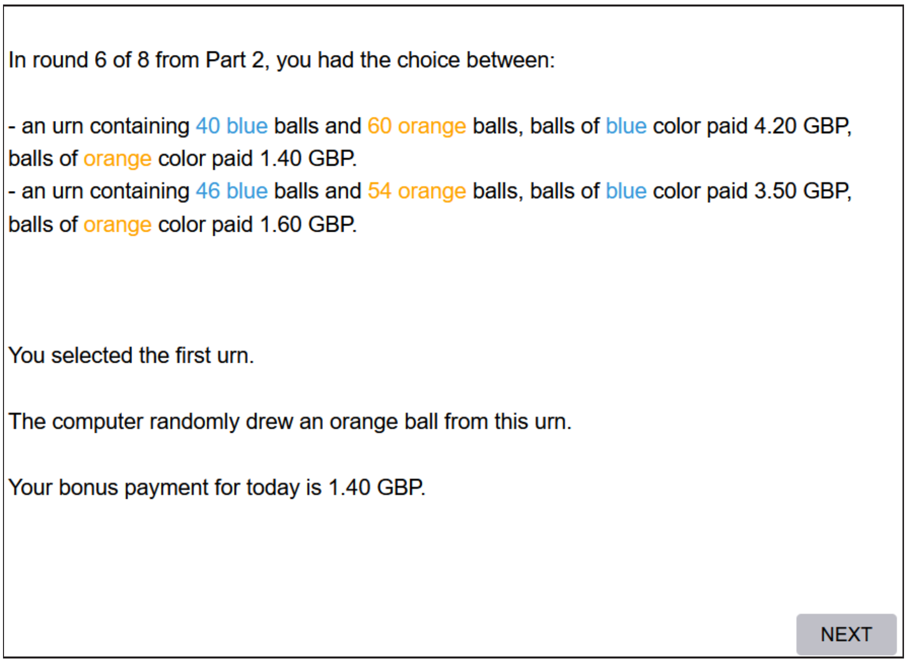

Fig. A.10 Screen 9 online experiment: Payment feedback for participant selecting lottery a in preference pair $(2,10)$.

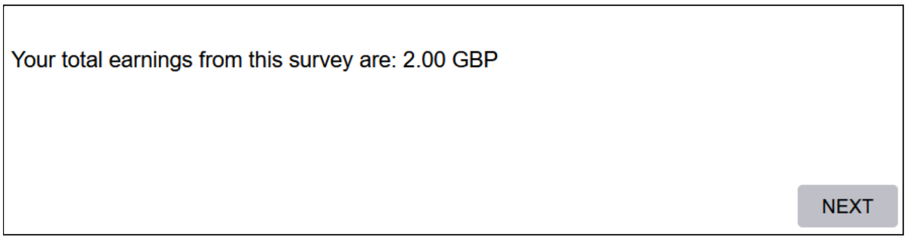

Fig. A.11 Screen 10 online experiment: Payment information.

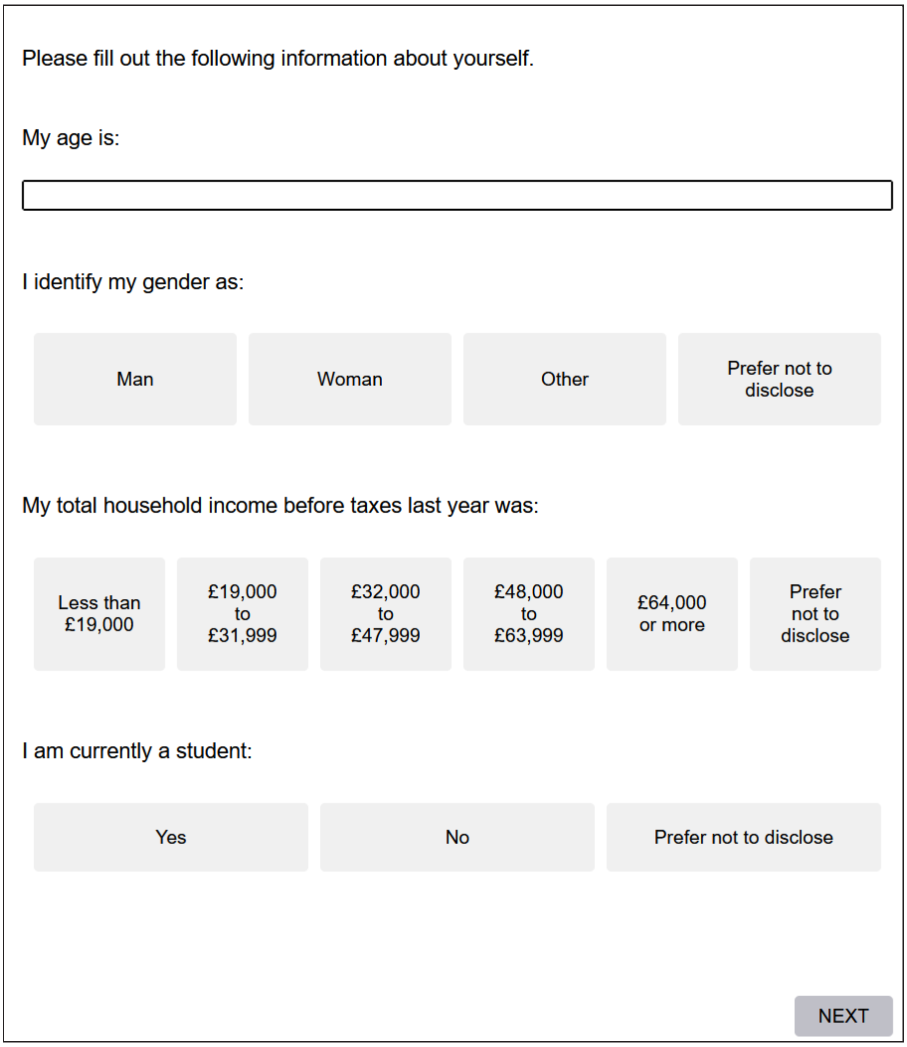

Fig. A.12 Screen 11 online experiment: Final questionnaire part 1.

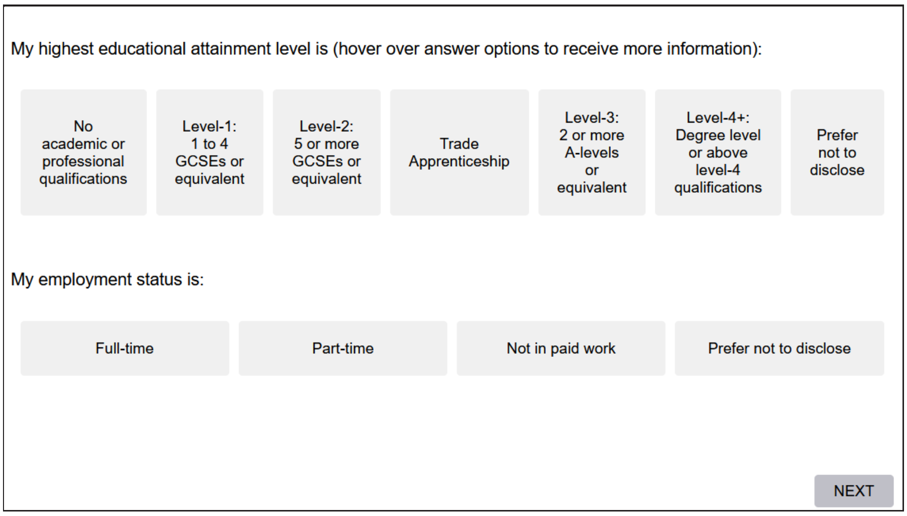

Fig. A.13 Screen 12 online experiment: Final questionnaire part 2.
